# Lactoferrin: neuroprotection against Parkinson's disease and secondary molecule for potential treatment

**DOI:** 10.3389/fnagi.2023.1204149

**Published:** 2023-09-05

**Authors:** Furkan Eker, Ecem Bolat, Burcu Pekdemir, Hatice Duman, Sercan Karav

**Affiliations:** Department of Molecular Biology and Genetics, Çanakkale Onsekiz Mart University, Çanakkale, Türkiye

**Keywords:** lactoferrin, Parkinson's disease, neuroprotection, heparan sulfate proteoglycans, dopaminergic cells, MPTP

## Abstract

Parkinson's disease (PD) is the second-most common neurodegenerative disease and is largely caused by the death of dopaminergic (DA) cells. Dopamine loss occurs in the substantia nigra pars compacta and leads to dysfunctions in motor functions. Death of DA cells can occur with oxidative stress and dysfunction of glial cells caused by Parkinson-related gene mutations. Lactoferrin (Lf) is a multifunctional glycoprotein that is usually known for its presence in milk, but recent research shows that Lf is also found in the brain regions. 1-Methyl-4-phenyl-1,2,3,6-tetrahydropyridine (MPTP) is a known mitochondrial toxin that disturbs the mitochondrial electron transport chain (ETC) system and increases the rate of reactive oxygen species. Lf's high affinity for metals decreases the required iron for the Fenton reaction, reduces the oxidative damage to DA cells caused by MPTP, and increases their surveillance rate. Several studies also investigated Lf's effect on neurons that are treated with MPTP. The results pointed out that Lf's protective effect can also be observed without the presence of oxidative stress; thus, several potential mechanisms are currently being researched, starting with a potential HSPG–Lf interaction in the cellular membrane of DA cells. The presence of Lf activity in the brain region also showed that lactoferrin initiates receptor-mediated transcytosis in the blood–brain barrier (BBB) with the existence of lactoferrin receptors in the endothelial cells. The existence of Lf receptors both in endothelial cells and DA cells created the idea of using Lf as a secondary molecule in the transport of therapeutic agents across the BBB, especially in nanoparticle development.

## 1. Introduction

Parkinson's disease (PD) is a multifactorial disease that occurs as a result of factors such as genetic heredity, aging, and environmental factors (Ozansoy and Başak, 2012). PD commonly occurs after the age of 50, and the frequency increases between the ages of 60 and 90 (Poewe et al., 2017). It generally leads to movement disorders such as rigidity, tremor, and postural instability (Balestrino and Schapira, 2020). The strong neuropathological characteristics of PD can be related to the Lewy bodies (LBs) that are dopaminergic neuronal intracytoplasmic inclusions (Ozansoy and Başak, 2012; Meade et al., 2019). One of the main indicators of PD development is the death of dopaminergic neurons in the substantia nigra at the basal ganglia (Tysnes and Storstein, 2017). The death of dopaminergic cells causes a disturbance in the dopamine flow in the related region, thus affecting muscle construction and harassing the coordinating movement. Compromised signaling in the region leads to the physical symptoms of PD (Parent and Parent, 2010; Fahim et al., 2013).

### 1.1. Iron in the central nervous system

Iron is an essential molecule with a role in several functions such as cellular respiration and oxygen transport. In the central nervous system (CNS), iron has functions such as neurotransmission, gene expression, and mitochondrial electron transport (Benarroch, 2009). Iron homeostasis is important in the brain's metabolism; thus, it must be tightly controlled and stabilized (Nnah and Wessling-Resnick, 2018). In the aging process, iron starts to accumulate in specific regions of the brain (Benarroch, 2009). In PD patients, iron accumulation is observed in the substantia nigra (Sokolov et al., 2020). In return, a high amount of iron increases oxidative stress, which leads to the disruption of dopaminergic neurons, and the cell goes into apoptosis. The dopaminergic neurons in the substantia nigra contribute to dopamine secretion in the nigrostriatal pathway and are highly disrupted in PD patients (Huang et al., 2017). The abnormal iron levels increase the ratio of the Fenton reaction. The reaction is carried out by the spontaneous reduction and oxidation of iron by hydrogen peroxide, resulting in the formation of hydroxyl radicals with oxygen species (ROS) (Cornelis et al., 2011). The hydroxyl radicals are dangerously reactive and can cause damage to many types of macromolecules without any enzymatic protection.

### 1.2. Glial cells and their disruption with alpha-synuclein

Glial cells are non-neuronal cells that are found in the nervous system (Gosselin et al., 2010). They are abundant in the nervous system and have complex interactions with the nerve cells depending on their subclass. Microglia are macrophages of the CNS, and their activated form produces proinflammatory molecules such as cytokines and ROS (Huang et al., 2017). They function as immune cells in the brain and are responsible for the synthesis and release of lactoferrin, anti-inflammatory, and proinflammatory compounds (Song et al., 2018; Naidu et al., 2021). They also influence the development and survival of neurons by producing brain-derived neurotrophic factors (Cardinale et al., 2021). Astrocytes, another type of glial cell found in the CNS, are responsible for the homeostasis of the brain microenvironment. Their function can be extended by balancing oxidative stress with glutathione (GSH) synthesis, releasing neurotrophic factors, and so on Joe et al. (2018). Even though glial cells can initiate immune responses, their overexpression might contribute to the degeneration process of the neurons (Dringen, 2005). There are specific types of molecules that are released from damaged and dying cells, which are known as damage-associated molecular patterns (DAMPs) (Takahashi and Mashima, 2022). Their presence activates the initiation of immune-like responses in the CNS, thus inducing inflammatory responses (Roh and Sohn, 2018).

Alpha-synuclein (α-Syn), a type of DAMP, is a small (140 amino acid length) soluble protein that influences neurotransmitter release and regulates the activity of synaptic vesicles (Stefanis, 2012; Meade et al., 2019; Cardinale et al., 2021). In the brain, they are mainly found in presynaptic neurons that are responsible for releasing transmitters to synapses (Lykkebo and Jensen, 2002; Yavich et al., 2004). During PD, α-Syn is accumulated in the neuron's cell body, rather than the nerve terminal, and causes abnormalities (Lykkebo and Jensen, 2002). The accumulated α-Syn molecules are released into the environment after the death of the cell and are thought to be responsible for damage to glial cells and causing their dysfunction (Takahashi and Mashima, 2022). The unusual uptake of α-Syn by astrocytes consistently induces their activation and creates neurotoxicity with inflammation (Chavarría et al., 2022).

Lactoferrin (Lf) is a multifunctional glycoprotein with approximately 80 kDa molecular mass and 700 amino acids (Iglesias-Figueroa et al., 2019). Lf is equipped with two alpha-helix-connected lobes, which are N and C. Each lobe can bind to a metal ion, such as Cu^+2^, Zn^+2^, Mn^+3^, and Fe^+3^ (ferric ion) (García-Montoya et al., 2012). Lf can be found in milk and is followed by mucus, white blood cells, and saliva in mammals (Karav et al., 2017). Lf's presence in different regions of the body, especially in the brain, can be explained by its antioxidant, antibacterial, and antiviral effects (Le Parc et al., 2017). These activities, especially the antioxidant characteristic of Lf, are mostly mediated by its ability to bind iron with high affinity. Immune-like activities of microglia can be observed under iron accumulation by the simulation of lactoferrin synthesis. Specifically in the area where DA neurons are found, Lf is highly expressed as a brain's alternative way to fix iron accumulation and stop ROS damage. Furthermore, Lf is observed in PD patients (especially in tears and saliva) more than in control groups, and using this fact as a PD marker is significant for many studies (Sokolov et al., 2020). At the same time, PD-related inflammation leads to the secretion of Lf by active microglia. Lf has been shown to have anti-inflammatory properties (Bolat et al., 2022). Not so surprising, Lf can decrease the number of proinflammatory cytokines that are secreted from glial cells under certain circumstances (Xu et al., 2019). In other words, Lf also accumulates in the area that exhibits PD-related inflammation and shows a positive effect.

### 1.3. Mutations in glial cells and their contribution to PD

**Figure 1:**
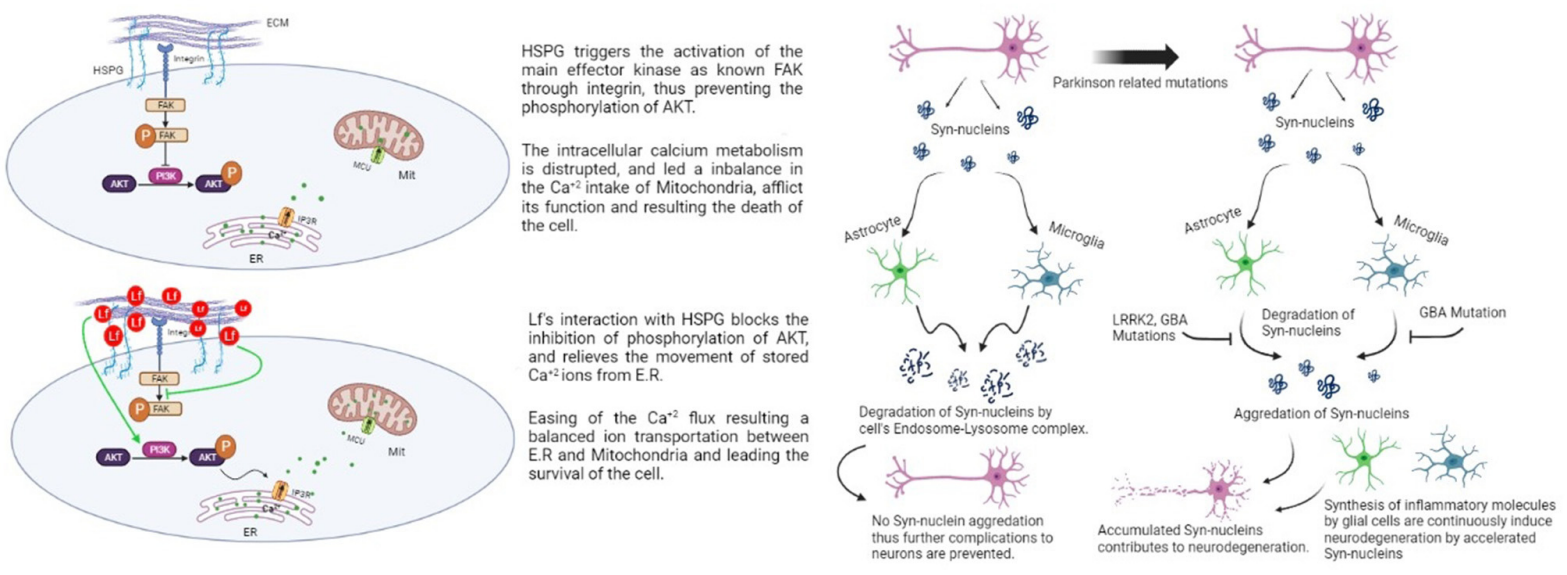
Effect of aggregated synuclein with mutated genes and lactoferrin's potential protective effect on dopaminergic cells via HSPG (Rousseau et al., 2013; Sokolov et al., 2020).

Familial Parkinson's disease occurs as a result of mutations in six different genes, namely SCNA, Parkin, DJ-1, PINK1, LRRK2, and ATP13A2. The most common polymorphisms are SCNA and LRRK2, which were confirmed as risk factors for disease (Ozansoy and Başak, 2012). Mutations related to PD cause critical dysfunctions in glial cells. Damaged neuron cells secrete α-Syn in the environment and activate glial cells by receptor binding. The received α-Syn has been exposed to lysosomal degradation in both microglia and astrocytes. The fractionation system of α-Syn inhibits the accumulation of this protein, but several proteins impose PD-related mutations that disrupt this system (Figure 1). In astrocytes, leucine-rich repeat kinase 2 (LRRK2) and glucocerebrosidase (GBA) mutations cause inhibition of the degradation of α-Syn and the signals of oxidative stress. The GBA gene is located in the lysosome and codes for lysosomal enzymes, thus leading to problems in protein degeneration and increasing exosomal release of α-Syn (Booth et al., 2017; Tremblay et al., 2019). According to these findings, a study reported that PD patients with the GBA mutation had substantial cognitive dysfunction (Chahine et al., 2013). When the mutation occurs in astrocytes, it induces mitochondrial imbalance and cell damage, eventually contributing to neurodegeneration (Ramos-Gonzalez et al., 2021). GBA mutations cause the same type of dysfunction in microglia (Mullin, 2021).

After all, it can be concluded that there is a great possibility in PD patients to observe a non-functionality in glial cells. This possibility and problem not only make glial cells an unfavorable treatment agent for PD patients but also make it important to understand and study the problems that affect glial cells. Having a more clear and broad view of glial cells in PD can contribute to comprehending Lf synthesis from microglia under high iron levels and the possible mechanisms behind it. Revealing the unanswered questions in the Lf-DA cell interaction with this approach might contribute to studies that aim to decrease DA cell deaths. Preventing the iron accumulation or the death of DA cells can both give the literature a new perspective on the current case and decrease the progress of the disease crucially with new information about glial cells.

## 2. Lactoferrin for dopaminergic cell protection and PD treatment

Lf has ROS modulator properties by inhibiting the Fenton reaction that causes damage to DA cells. Lf can protect DA cells under the oxidative stress created by this reaction in apo (iron-free) form. In addition to protecting dopaminergic neurons by affecting iron metabolism, LF enhances the expression of brain-derived neurotrophic factor (BDNF) through the extracellular signal-regulated protein kinase (ERK)—cAMP response element-binding protein (CREB) pathway and HIF-1-dependent mechanisms to reverse the movement disorders caused by PD. For example, an animal study performed with postnatal piglets was performed to observe Lf's effect on neurodevelopment (Chen et al., 2015). BDNF plays an important role in both memory and learning. The research confirms that Lf increases the BDNF signaling pathway. Through this, the Lf-treated group showed increased levels of BDNF by the phosphorylation of several transcription factors (such as CREB) due to activated pathways. The increased expression led to neuronal and cognitive function improvements in the Lf group.

For the potential use of Lf for PD treatment, a mouse model has been used (Xu et al., 2019). One of the aims of the study was to find the Lf's effect on PD-like motor dysfunctions in exposure to dopamine neurotoxin. When certain movement features were compared, such as distance and climbing grid, the Lf-treated toxin group had higher numbers than the untreated toxin group. They indicated that human Lf exposure decreased PD-related symptoms and can be used as an alternative, safe drug, especially for movement disorder issues.

Last but not least, Lf receptors can be found in high abundance in the brain's capillary endothelial cells and neurons. The accessibility of Lf into the brain region indicates that Lf can cross the BBB and reach neural tissues; thus, it can play an important role in the delivery of therapeutic molecules. Therefore, Lf carries a huge potential as a main or secondary molecule to be used in brain-targeting delivery systems, either directly or indirectly (Huang et al., 2007, 2010; Kopaeva et al., 2019).

Several reviews explained that both microglia and astroglia contribute to PD by negatively affecting the balance of iron levels (Huang et al., 2017; Song et al., 2018; Kam et al., 2020). Therefore, iron chelators are highlighted as an alternative agent for decreasing excess iron. Chelators are capable of forming bonds between a metal ion and a ligand (Liu et al., 2010). Some of these chelators have a high affinity for iron, so they prevent the iron's contribution to the Fenton reaction as it cannot stay in free-roaming form. As mentioned, microglia are stimulated to synthesize the iron chelator molecule Lf at high levels of proinflammatory cytokines, oxidative stress, and most importantly iron accumulation (Vincent et al., 2001). Synthesis of Lf in high iron level conditions promises a huge solution to the issue that we pointed out in the glial cell disruption part. These findings and the connections that were created from them gave the idea of the usage of Lf as a neuroprotective agent, and related studies started to be performed.

1-Methyl-4-phenylpyridinium (MPP^+^) is a type of dopamine neurotoxin that induces Parkinson's disease and causes movement disorders (Tetrud and Langston, 1989; Fuller and Hemrick-Luecke, 1990). MPTP, an inactive form of MPP^+^, is selectively toxic to the substantia nigra and accumulates in the neurons (Langston et al., 1984). Unlike MPTP, MPP^+^ cannot cross the blood–brain barrier. Therefore, MPP^+^ is taken in by the dopaminergic nerve endings with the help of the dopamine uptake system, taking advantage of its structural similarity with dopamine. When taken inside, it damages nerve cells by disrupting the NADH dehydrogenase metabolism of mitochondria and the complex I activity of the electron transport chain (Tetrud and Langston, 1989).

An animal study was performed to understand the Lf's protective effect against neurons in the midbrain region. The study showed that Lf had a positive effect on neurons that are treated with MPP^+^ neurotoxin. In the treatment, both apo (iron-free) and halo (iron-saturated) forms of Lf were used and they both successfully showed a protective effect. This means that Lf's neuroprotective effect against MPP^+^ was not dependent on its iron bind capability (chelation). MPP-treated environments induced Lf levels in the area, simultaneously with Lf mRNA and receptor levels. Even though Apo Lf decreased neuronal iron accumulation with a visible iron chelation effect, the main effect presumably was either restoration of mitochondrial function or protection of neurons from toxicity-induced apoptosis (Wang et al., 2015).

In another similar research, pretreatment of human Lf (hLF) in an MPTP-mediated mouse model of the nigrostriatal system was performed. The treatment of hLf led to the recovery of motor functions and exploratory behaviors in mice. Plus when the tyrosine hydroxylase (TH^+^ cells found at substantia nigra, a marker for dopaminergic cells in the central neuron system) (Nagatsu et al., 2019) quantities were determined and compared, there was a slight increase in the number of TH^+^ cells in Lf-treated mice (Kopaeva et al., 2021). The apoptosis route might be possible as Lf can promote cell apoptosis by regulating signaling molecules such as poly(ADP-ribose) polymerase (PARP) (Li and Guo, 2021).

Parkinson-related cell death might occur with a different type of cell death, and Lf might not only interfere with the apoptosis route. One of the first studies investigated the transcytosis of Lf in BBB from the perspective of tumor necrosis factor-α (TNF-α) (Fillebeen et al., 1999). It was observed that TNF-α increases Lf transcytosis in the BBB. TNF is a type of cytokine that alters diverse cellular activities, such as cellular death, proliferation, or survival (Wang and Lin, 2008). TNF is secreted not only from inflammatory cells but also from astrocytes and microglial cells. PD-related inflammation causes both the secretion of TNF and lactoferrin, and so this might explain the TNF-α influence on Lf-mediated transcytosis in BBB. One of the examples worth investigating is an animal study (Li et al., 2021). Lactoferrin-modified nanodots were used to investigate MPTP exposure in mice. During the experiment, necrosis-mediated cell death decreased from 23.3 to 12.5% in the Lf-nanodot group. Even though necrosis-mediated cell death in PD from the Lf perspective is not well established, it might be a good approach to address the current issue.

Methamphetamine (METH) is a neurotoxic drug that negatively alters the dopaminergic pathways in the CNS (Ares-Santos et al., 2013). It is mentioned that after repeated exposure to METH in mice, a decrease in TH^+^ cells was observed. To investigate the Lf's effect on METH-based negative mitochondrial changes and autophagy cell death, an *in vitro* study was performed (Ryskalin et al., 2021). The results indicate the Lf's positive effect on both mitochondria and the autophagy status of the cell by decreasing cellular death and relieving autophagy-related proteins from autophagy compartments. Lf's potential neuroprotection effect against METH might be one of the many examples. Approaching PD–Lf interaction from a cellular death mechanism perspective might directly answer several underlying mechanisms mediated by Lf in future, but many investigations are still required for clear evidence.

In addition to so many studies on lactoferrin, data on the effects of lactoferrin on proinflammatory cytokines, iron metabolism, and MPTP are insufficient, as can be seen in the Table 1. Therefore, more studies on lactoferrin should be carried out in these areas to gain more information about both Lf and PD.

**Table 1:** Studies about lactoferrin's effect on Parkinson's pathogenesis.

**Pathogenesis of PD**	**Lactoferrin's effect**	**Study type (used cell lines)**	**Form of lactoferrin**	**References**
Neuronal death in substantia nigra	Neuronal death decreased and apoptosis decreased.	*In vivo*, used rat *In vivo*, used mice	Recombinant human lactoferrin/human lactoferrin	(Rousseau et al., 2013; Wang et al., 2015; Zakharova et al., 2018; Xu et al., 2019; Liu et al., 2020; Kopaeva et al., 2021)
Fe accumulation in substantia nigra	Fe numbers and metabolism are normalized in the region	*In vivo*, used mice *In vitro*	Human lactoferrin	(Wang et al., 2015; Xu et al., 2019; Gholkar et al., 2020; Liu et al., 2020)
Abnormal synthesis of proinflammatory cytokines	A drop in the inflammatory cytokine numbers	*In vivo*, used mice *In vivo*, used rat	Human lactoferrin	(Xu et al., 2019)
Oxidative stress exposure	Oxidative stress levels are decreased and/or antioxidant enzymes are increased	*In vivo*, used rat *In vivo*, used mice	Recombinant human lactoferrin/human lactoferrin	(Rousseau et al., 2013; Zakharova et al., 2018; Xu et al., 2019)

The potential mechanism behind Lf's protective effect against MPTP was discussed in an animal midbrain culture study with hLf treatment (Rousseau et al., 2013). In the research, Lf-treated dopaminergic cells are exposed to MPP^+^. The DA cells are successfully protected from the effects of the mitochondrial toxin. They conclude the results by stating that Lf might be targeting mitochondria as a downstream target. A decrease in ROS levels is also determined in the DA cells. The findings demonstrated that Lf acts as an iron chelator when there is oxidative stress but also shows another potential effect in the absence of oxidative stress. It shows a direct neuroprotective effect in DA cells by regulating a specific pathway for calcium shuttling. Lf's regulating effect takes place by binding heparan sulfate proteoglycans (HSPGs) that are found on the surface of the DA cells. HSPGs have a role in several pathways in dopaminergic cells (Figure 1), such as being an essential modulator in diencephalon spinal mechanisms of DA cells (Kastenhuber et al., 2009). They created a representation of the consequences of HSPG-Lf binding. Protein kinase B (AKT) has essential functions for the survival of dopaminergic cells, such as regulating cell proliferation, apoptosis, and cell migration (Brazil et al., 2004; Bao et al., 2012).

The Ca^+2^ flux between the endoplasmic reticulum and mitochondria is mediated by the AKT pathway. AKT phosphorylation mediates the entrance of the Ca^+2^ ions into the intercellular space of the cell via the SOC channels (mediates the Ca^+2^ movement from extracellular to intracellular) (Selvaraj et al., 2012). These findings could indicate and explain the effect of the Lf treatment on the dopaminergic cells. Lf possibly reduces the activation of facial adhesion kinase (FAK), a kinase that blocks the AKT-dependent signaling pathway by inhibiting its phosphorylation. Hereby, Lf binds to HSPGs on the cell surface, relieves the block of the AKT pathway, and enhances AKT phosphorylation to support the regulation of Ca^+2^ levels within the cell (Rousseau et al., 2013).

A highlighted study that used the first model of *Caenorhabditis elegans*, a nematode chosen for having a similar somatic nervous system to humans with nearly 80% similarity to human genes by having orthologs, were performed for observation the HSPG effect in alpha-synuclein toxicity (Chen et al., 2022). With recombinant human wild-type alpha-synucleins, alpha-synuclein-preformed fibrils were generated and digested by *Caenorhabditis elegans*. With the RNAi-mediated knockdown test of the enzymes involved in HSPG synthesis, it has been revealed that this toxicity mediated by synuclein fibrils depends on the HSPG pathway. This first *in vivo* observation about alpha-synuclein toxicity shows that the toxicity starts in the gut and moves into the brain. The fact that lactoferrin has assorted roles in the gut and binding capabilities with HSPG makes this research promising evidence of a potential protective effect of Lf. To add more, it should be mentioned that there are almost no studies on lactoferrin's effect on MPTP in human dopaminergic cell lines. The observed recovery in MPTP rat models makes lactoferrin a good potential choice in future human studies, so further research must be performed.

Last but not least, research was performed on mature rats by injecting lipopolysaccharide to analyze memory impairment. For observation, some groups received Lf treatment. The inflammatory cytokine levels significantly decreased after Lf treatment, which demonstrates Lf's anti-inflammatory effect. It is stated that Lf's effect on memory impairment might be explained by its neuroprotective effect by increasing the neurotrophic factor level back to normal (Madi and El-Saka, 2018).

## 3. Usage of lactoferrin as a secondary molecule for curcumin delivery in Parkinson's disease

Protein accumulation triggers death and damage formation in neurons (nerve cells) through many different systems such as oxidative stress and neuroinflammation. Currently, using therapeutic molecules such as curcumin as an alternative in the treatment of neurological and neurodegenerative diseases is shown as an effective strategy (Yavarpour-Bali et al., 2019).

Curcumin is found in the dietary spice turmeric, that is, a rhizomatous herbaceous perennial plant (*Curcuma longa*) of the ginger family (Anand et al., 2007; Yavarpour-Bali et al., 2019). Curcumin possesses various pharmacological effects such as antioxidant, anticancer, anti-inflammatory, and neuroprotection (Del Prado-Audelo et al., 2019; Nasery et al., 2020). Despite the promising properties of this herb, it has poor bioavailability, which means low absorption due to a high rate of metabolism (Hewlings and Kalman, 2017; Del Prado-Audelo et al., 2019). Although several strategies increased the solubility and bioavailability of curcumin during the treatments, the high rate of metabolization still caused problems (Kuo and Tsao, 2017). Therefore, one of the most common approaches to this issue is using nanoparticles as a transport method (Wilczewska et al., 2012). These nanoparticles are used to increase the dispersion of hydrophobic molecules and improve the poor water solubility of therapeutic agents. Curcumin encapsulation in nanoparticle areas improves the chemical stability of curcumin and protects its degradation and bioavailability (Bollimpelli et al., 2016; Microbiota et al., 2019; Panaro et al., 2020). Nanoparticles can solve the ineffectiveness of bioavailability (Rakotoarisoa and Angelova, 2018), but the specificity problem remains. As a result, several modifications are made in order to create an efficient complex for delivery.

Lactoferrin receptors are present in the blood–brain barrier and allow their passage through receptor-mediated transcytosis. With the data that DA neurons also possess Lf receptors, especially with increased levels in disease-resistant cells, nanoparticles for curcumin delivery can be modified with Lf to solve the specificity issue in this case.

For instance, rotenone is a flavonoid-induced neurotoxicity in dopaminergic cells; thus, it can cause oxidative damage and cellular death with long-term exposure (Testa et al., 2005; Sai et al., 2008). A research were performed in a model of neurotoxicity mediated by rotenone to test the curcumin delivery. In result, the curcumin delivery were successfully mediated by loading curcumin loaded into Lf-modified nanoparticles and displays a positive effect on protecting DA cells against Rotenone exposure by detecting a significant decrease in ROS levels (Bollimpelli et al., 2016). Furthermore, a decrease in alpha-synuclein and an increase in TH^+^ cells were observed in the results. As a result, uptake in nerve cells, intracellular localization, and target specificity of curcumin encapsulated with lactoferrin molecules were ensured (Anand et al., 2007; Hewlings and Kalman, 2017; Del Prado-Audelo et al., 2019). Research implies that PD patients have an increased amount of lactoferrin receptors in their cells at the mesencephalon. The research found data that Lf nanoparticles successfully decreased ROS levels in mucus to higher levels than sol curcumin with specialized specificity. To conclude, they suggested that lactoferrin has a huge potential for being a supportive compound for treatment agents that are used in PD patients, especially in the nanoparticle area (Bollimpelli et al., 2016).

## 4. Conclusion

PD is a multifunctional disease, and its neuropathological specialty can be explained by Lewy bodies in dopaminergic neurons. The death of the nerves in the region of the substantia nigra pars compacta and the loss of the DA cells cause movement disorders. Iron metabolism plays a crucial role in it. Lactoferrin, a multifunctional protein with iron chelation ability, plays an important role in stabilizing essential iron metabolism in both extracellular and intracellular systems. The potential scenario, where the microglial cells readily synthesize lactoferrin in the parkinsonism exposure, creates a huge area for lactoferrin to play its part. From the perspective of mutated genes and dysfunctional glial cells, lactoferrin can be used to relieve Parkinson-mediated oxidative stress. Several studies mediated by the dopamine neurotoxin MPP^+^ suggest mechanisms for Lf's potential neuroprotection and increased survival of DA cells with HSPG interaction. Last but not least, due to the wide spectrum that Lf possesses in the brain, Lf can also become a supportive molecule for the therapeutic agent against PD. Lf can mediate receptor-mediated transcytosis, increase BBB permeation, and bind to the DA cells that are rich in Lf receptors, thus increasing the specificity and ensuring the safety of the therapeutic agent's route. The lack of data and research about Lf's potential neuroprotection against PD puts this subject in a substantial position when the current data are observed.

## Author contributions

SK organized the general content of the manuscript. FE and EB were responsible for general editing and organizing the authors as well as the contributions to sections. BP and HD contributed to one section of the manuscript. All authors contributed to the manuscript and approved the submitted version.
